# Ten simple rules for getting started with knowledge mobilization

**DOI:** 10.1371/journal.pcbi.1012888

**Published:** 2025-04-04

**Authors:** Rime Diany, Laura K. Fitzgibbon-Collins, Sarah A. Gagliano Taliun

**Affiliations:** 1 Faculty of Medicine, Université de Montréal, Montréal, Québec, Canada; 2 Department of Medicine, Division of Geriatric Medicine, Schulich School of Medicine & Dentistry, University of Western Ontario, London, Ontario, Canada; 3 Department of Kinesiology, University of Western Ontario, London, Ontario, Canada; 4 Research Centre, Montréal Heart Institute, Montréal, Québec, Canada; 5 Department of Medicine, Faculty of Medicine, Université de Montréal, Montréal, Québec, Canada; 6 Department of Neurosciences, Faculty of Medicine, Université de Montréal, Montréal, Québec, Canada; Dassault Systemes BIOVIA, UNITED STATES OF AMERICA

## Introduction

You acquired funding, data access, and computational resources. You developed and ran various computational models to test your hypothesis, and your team is ready to share your results. What is next? It is probably writing a manuscript for a scholarly journal or presenting your findings at a scientific meeting. However, dissemination of computational biology research can take many forms that extend beyond the conventional journal-based metrics and conference presentations most academics are accustomed to. Knowledge mobilization (KM) is a form of dissemination that involves transferring research output beyond traditional academic settings to a targeted audience such as a patient-facing population or the general public.

Although the concept of KM is not usually addressed in academia and is not a standard component of formal education in computational research, it is clear that the field of computational biology is exponentially growing beyond the current capacity to effectively translate these findings into best practices. Therefore, it is essential to provide a systematic approach in developing KM material for experts in this field. The capacity building for bioinformatics in Latin America (CABANA) project, for instance, was designed to strengthen bioinformatics capacity. The CABANA group identified a selected cohort of professionals (faculty members, research scientists, and students) to implement KM changes [1]. We have intended that the content of the current manuscript applies to individuals across diverse fields, but we have provided specific applications and examples to the field of computational biology.

Sharing academic knowledge in a way that is accessible and tailored to a broader set of individuals is crucial for translating important, yet challenging, concepts into meaningful pieces of information for the general public. Ineffective KM or a lack of KM leads to misinformation, and to limited knowledge or understanding of the factors surrounding health conditions and diseases [2]. The interpretation of scientific output by the general population is especially important in computational fields where research output may not be readily interpretable by the public. Furthermore, education is an important part of medical care: well informed patients are patients who are in a much better position to take care of themselves. A study assessing 5405 participants from the general population, found that knowledge of genetics was positively associated with willingness to use such knowledge for personal health management; however, genetic literacy in the general population is poor [3]. Therefore, it is important to present KM output in a manner that allows for informed uptake by the intended audience.

**Fig 1:**
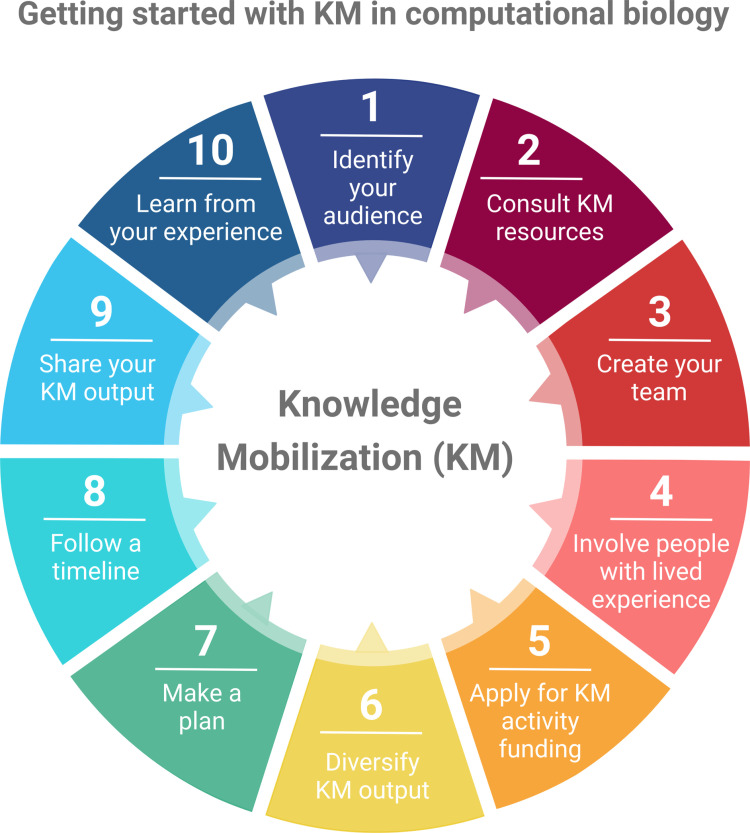
Summary of rules for getting started with knowledge mobilization. Created in biorender com.

KM in the academic setting is not typically a standard part of scientific training, and therefore translating research into accessible formats for use by the general public can be daunting to embark upon. Due to the lack of existing KM applications in the computational biology field, we have provided a few rules (**Fig 1**) to get started.

## Rule 1: Identify your audience

Knowing your intended audience will guide subsequent steps. Determine who you want to reach with your form of dissemination. The knowledge users could be the general public, students (high school or post-secondary), patients, care partners, clinicians, or another targeted population.

Once an intended audience has been outlined, you can develop corresponding KM strategy objectives. KM objectives have been categorized by Ziam et al. 2024, which include the exposure and dissemination of knowledge, intended change of attitudes/raise awareness, inform/influence decision-making, increase expertise/confidence to improve practices, and foster collaborative partnerships [4, 5]. Consider how each of the aforementioned objectives are applicable and can be outlined in your computational biology project by your KM team (see Rule 3).

Identifying the intended audience and corresponding objectives will greatly influence the content and format of the KM planning and delivery. For computational genetics work, for example, it is important to also consider the genetic literacy of the target audience: it will determine the contextualization needed for each concept mentioned. Furthermore, the type of audience will determine the most effective way to transmit the knowledge.

## Rule 2: Consult KM resources

Research-intensive institutions often have a dedicated team of individuals to offer KM support and/or a collection of tools and resources available to aid in translating knowledge. Getting help from a person with extensive KM experience will keep you moving forward as you embark upon your KM activities. Begin by asking who you can reach out to at your Research Centre or Institution. Most Canadian universities, for instance, have a research impact hub and/or a KM unit to encourage research outputs with a broader reach. Reach out to colleagues at your institution who have led or participated in KM activities in the context of their projects. If you find that resources are lacking at your institution, speak with your department chair. KM needs to be made a priority, particularly in computational research, to begin to close the gap between computational research findings and eventual translation. Research funding agencies may also have people, material and financial resources to assist in KM (see Rule 5).

Vulgarizing computational biology results can be a challenge, and seeking a consultant who specializes in dissemination of niche fields can be helpful. Returning to our opening example of computational genetics, the Wellcome Connecting Science’s website *yourgenome* (https://www.yourgenome.org/) is one example of a genomics education portal with resources available for children, adults and educators [6].

## Rule 3: Create your team

Choose to carry out your KM activity in a diverse team setting in terms of expertise and experience. There are efforts to encourage bidirectional collaborations between the computational and experimental biologists to bring together these complementary skillsets, knowledge and experiences to devise, analyze and interpret scientific questions that can be investigated in part using computational biology approaches [7]. These varying perspectives will only benefit the resulting KM output. Consider also including a KM expert on your team (see the previous rule) as well as at least one individual with lived experience (see the following rule).

## Rule 4: Involve people with lived experience

Individuals with lived experience should be involved in all aspects of the project, from its inception to KM. Their role should not be limited to a study participant as is the default in computational biology research. Partnering with people who have lived experience ensures that the KM output is relevant, relatable and effective. This partnering can be done in multiple ways, including through surveys, in-depth interviews, and focus groups [8].

The co-creation of KM with its end-users is an efficient way to ensure your approach meets your audience’s needs. Collaborating with knowledge users who have different levels of literacy on a topic crucial to your project (e.g., genetics, statistics, biochemistry, etc.) is a way to ensure that the format and content of the dissemination tool is adapted to the intended audience. It is also a way to ensure that the message is conveyed accurately while being culturally relevant [8].

Non-profit organizations can provide recommendations and guidance for recruiting individuals with lived experience [9]. Organizations supporting health research are a valuable resource for connecting individuals with lived experience of particular conditions (Alzheimer Society, Cancer Society etc.) to specific research fields and topics. In the UK and in Canada, for example, there are online platforms that allow researchers to find members of the public who are interested in getting involved in research (the NIHR’s People in Research: https://www.peopleinresearch.org, and the CIHR’s Strategy for Patient-Oriented Research: https://cihr-irsc.gc.ca/e/41204.html).

## Rule 5: Apply for KM activities funding

Bridging from the previous rule, various funding bodies allow for individuals with lived experience to actively be a part of grant submissions as a co-applicant or collaborator within the research team. In the US, the Education Projects Resource Grant of the National Institutes of Health, allows for the development of ways to disseminate discoveries. In Canada, funding opportunities directly aimed at supporting KM activities include the Canadian Institutes for Health Research’s Planning and Dissemination Grants program and the National Research Council of Canada’s Outreach Initiative grants program. This non-exhaustive list provides a sample of concrete examples of KM funding initiatives. Specific initiatives in the field of computational biology in North America and elsewhere, such as through the Chan Zuckerberg Initiative, the Gates Foundation and Wellcome Connecting Science among others, have aided in KM activities across the globe.

## Rule 6: Diversify KM output

Diversify the form of your KM output to appeal to individuals within your intended audience group who have different learning and retention styles. The options are vast, including online or hard copies of text-based, video-based or art-based forms and more. KM activities can be classified as theories, models, and frameworks to accomplish various goals, such as to train or educate, develop interrelationships, engage the intended audience, or support clinicians [5,10,11]. Within the area of computational biology, a variety of KM approaches are applicable depending on the context of the research and the intended audience. A summary of KM output types has been previously covered in a Ten Simple Rules article [12].

## Rule 7: Make a plan

Now it is time to assemble a KM plan. There are various publicly available resources for inspiration, which can be curated for any individual research project (**Table 1**).

**Table 1:** Examples of resources for planning or assessing KM activities.

Resource	Developer	Link	Purpose
Knowledge Translation Planning Template [13]	The Hospital for Sick Children	https://www.sickkids.ca/siteassets/learning/kt_planning-template_may2021.pdf	Plan for knowledge translation
Dissemination & Implementation Models in Health [14]	University of Colorado Denver	https://dissemination-implementation.org/tool/	Plan and select, as well as use and assess dissemination models
The Patient Education Materials Assessment Tool (PEMAT) [15]	Agency for Healthcare Research and Quality	https://www.ahrq.gov/health-literacy/patient-education/pemat.html	Assess understandability and actionability of (patient) education materials
Disseminating Results to the Public [16]	Vanderbilt Institute for Clinical and Translational Research	https://victr.vumc.org/disseminating-results-to-the-public/	Access multiple tool kits on knowledge dissemination

Identifying the problem or the gap in knowledge that will be addressed by your KM output is a good way to start your plan and then begin to contextualize this knowledge to your intended audience (see Rule 1). It is important to carefully identify the gap to be addressed and ensure it is adapted appropriately to your audience. Conducting a literature review to identify gaps and discussing your ideas with individuals who have lived experience (see Rule 4) can add a unique perspective to your work.

For example, let’s say that your research project assesses the role of loss-of-function variants in a specific set of genes in the development of a disease. The gap in knowledge to be addressed by the KM activity could be the lack of knowledge in the general population on the role genetics in the development of this disease. A resulting KM activity to fill this gap could be aimed at raising awareness of the genetic contributors to the disease of interest (e.g., development of infographic material, an interactive presentation at a high school, etc.). Alternatively, perhaps the knowledge gap is insufficient routine screening of this class of variants in clinical settings, and the target audience are screening policy makers and clinicians. In this case, the goal of the KM activity could be to inform and facilitate policy changes in hospitals regarding genetic screening, and the audience would be the pertinent decision makers and clinicians.

## Rule 8: Follow a timeline

Alongside the creation of your plan (see previous rule), decide upon a realistic timeframe to carry out the KM activity, given available resources and person-power.

While planning the timeframe, it is important to consider changes that can occur in the relationship between your audience and the information that you want to transmit, especially when the timeframe is significant. For example, new needs can arise or new ways to communicate with the target audience can emerge. A well redacted plan is also a plan that can accept a certain flexibility.

## Rule 9: Share your KM output

Congratulations! You have finished your KM output, and it is now ready to disseminate among your intended audience. Collecting data on the number of people reached (and their demographic characteristics) is one way to measure the potential impact of your KM tool.

## Rule 10: Learn from your experiences

Like paper or grant writing, KM development is an acquired skill that takes practice. Gather feedback from your audience and from your team throughout the whole process. Then, take the time to debrief as a team at the end of a KM project. Which aspects worked well? What would you have changed? Use this information to inform and improve your next KM activity.

## Conclusion

Implementing modes of research dissemination that foster KM beyond the traditional academic domain is essential to bridging the gap between computational models and meaningful uptake of knowledge. Here we provide ten simple rules to get started in KM activities with a focus on computational biology, but with transportability to other fields.
